# Deciphering Roles of Placental Endoplasmic Reticulum Stress in Complicated Pregnancies and Beyond: The Power of Mouse Models

**DOI:** 10.3390/cells15020096

**Published:** 2026-01-06

**Authors:** Hong Wa Yung, Yat Nam Yung, Graham J. Burton, D. Stephen Charnock-Jones

**Affiliations:** 1 Department of Obstetrics and Gynaecology, University of Cambridge, Cambridge CB2 0SW, UK; dscj1@cam.ac.uk; 2 Loke Centre for Trophoblast Research, University of Cambridge, Cambridge CB2 3EG, UK; gjb2@cam.ac.uk; 3 Gonville and Caius College, University of Cambridge, Cambridge CB2 1TA, UK; yny20@cam.ac.uk

**Keywords:** endoplasmic reticulum stress, placenta, pregnancy, fetal growth restriction, pre-eclampsia, animal model

## Abstract

**Highlights:**

**What are the main findings?**

- Transgenic mouse models confirm that placental ER stress-mediated loss of PI-3K-AKT signalling and bioactivity of angiogenic factors play key pathophysiological roles in fetal growth restriction and early-onset pre-eclampsia.
- A placental endocrine-specific transgenic model suggests that ER stress-mediated loss of placental signals results in maternal maladaptation to pregnancy.

**What are the implications of the main findings?**

- Targeting placental ER stress may provide a potential therapeutic intervention reducing complications of human pregnancy.
- Placental dysfunction has longer-term implications for maternal health than the duration of the index pregnancy.

**Abstract:**

Over a quarter of human pregnancies are associated with complications, including fetal growth restriction, pre-eclampsia and gestational diabetes. These are major causes of maternal and fetal morbidity and mortality, and also lead to a 3–5-fold increased risk of subsequent development of cardio-metabolic diseases. Although the mechanistic details remain elusive, a dysfunctional placenta is central to the pathophysiology of these conditions. The placenta ensures sufficient nutrient supply to the fetus without compromising maternal wellbeing. This balance is achieved by the secretion of large quantities of placental-derived peptide hormones into the maternal circulation. Consequently, the placenta is susceptible to endoplasmic reticulum (ER) stress, and we were the first to demonstrate the presence of ER stress in placentas from complicated pregnancies. The mouse placenta provides an ideal model for studying the impact of ER stress as it is composed of two distinct regions, an endocrine zone and a transport zone. Therefore, perturbation of placental endocrine function by ER stress can be generated without directly affecting its capacity for nutrient exchange. In this review, we summarise the current literature on how transgenic mouse models enhance our understanding of ER stress-mediated perturbation of placental endocrine function, and its contribution to the pathophysiology of pregnancy complications and life-long health.

## 1. Introduction

There are an estimated 200 million human pregnancies worldwide each year. Over 25% of these are associated with various complications, such as pre-term labour, fetal growth restriction (FGR) or small for gestational age (SGA), pre-eclampsia (PE) and gestational diabetes (GDM) [1,2,3]. These complications are major causes of immediate maternal and fetal mortality and morbidity. Moreover, epidemiological studies on postpartum women and their babies after complicated pregnancies reveal that the adverse impact on health goes beyond the duration of pregnancy. These women have a 3-to-5-fold increased risk of developing cardio-metabolic conditions such as hypertension, type II diabetes and obesity in later life [4,5].

Although the mechanistic details behind these pregnancy complications remain elusive, a dysfunctional placenta is widely recognised to be central to their pathophysiology. Many complications arise due to malperfusion of the placenta secondary to deficient adaptations of the maternal uterine vasculature to pregnancy. Malperfusion results in differing degrees of oxidative and endoplasmic reticulum (ER) stress, depending on its severity. At low levels of activation, the ER stress response is a homeostatic mechanism aimed at ensuring that protein synthesis, an energetically and metabolically demanding process, is matched to nutrient and oxygen availability and prevents the accumulation of toxic misfolded proteins. By contrast, sustained high levels of activation can lead to cell senescence, associated mitochondrial dysfunction and apoptosis. To investigate and understand the relative contributions of these pathways to placental pathologies, and to develop therapeutic interventions, multiple approaches have been employed. These include the use of traditional methods, including direct investigation of the molecular signatures and signalling changes in pathological placental tissues and ex vivo culture of placental samples and primary or immortalised placental cells. More recently, new state-of-the-art approaches have been adopted, such as human trophoblast and endometrial organoid cultures [6,7], multi-omics-based studies [8] and data-driven modelling [9]. Technological advances have also enabled the development of a micro-fluidic system such as placenta-on-a-chip [10,11] and multi-organs-on-a-chip models for disease modelling [12]. For example, a foeto-maternal interface organ-on-a-chip model has been used for studying pregnancy pathology and drug testing [13].

Although these approaches have undoubtedly accelerated research into understanding the pathophysiology of pregnancy pathologies, they fail to fully recapitulate the complex cellular interactions and communication among mother, placenta, and fetus in vivo. They fail to replicate, for example, the spatial and temporal interactions among various cell types at the maternal–fetal interface and display limited physiological fluid dynamics and buffering. In addition, they cannot model the different phases and entire duration of pregnancy, which can only be achieved in animal models. In this review, we focus mainly on how transgenic mouse models advance our understanding of placental cellular stresses, particularly endoplasmic reticulum (ER) stress, and their contribution to the pathophysiology of human complications of pregnancy. We will also discuss how placental-specific ER stress animal models can be used to test candidate molecular mechanisms that predispose women to increased risk of long-term poor health after a complicated pregnancy.

## 2. The Placenta Is Vulnerable to Cellular Stresses

The placenta is a temporary organ existing only at the time of pregnancy. It can be classified into four subtypes based on the histology of the maternal–fetal interface; haemochorial (e.g., mouse, rat, rabbit and human); endotheliochorial (e.g., cat and dog); synepitheliochorial (ruminants) and epitheliochorial (pig and horse). Placental size, shape and structure vary considerably among species, but in all its prime function is supporting fetal growth and development. Its activities include nutrient and gas exchange, removal of metabolic waste, immune protection and forming a selective barrier to prevent harmful substances reaching the fetus. In addition, the placenta facilitates physiological adaptations in the mother that help sustain pregnancy and lactation. These include increased cardiac output, liver enlargement, insulin resistance, increased maternal blood volume and maturation of the mammary glands. Thereby, the placenta supports fetal growth without compromising maternal wellbeing [14]. These maternal adaptations are largely orchestrated via placental synthesis and secretion of large quantities of proteins and hormones into the maternal circulation. For example, by term the human placenta produces approximately 1 g per day of placental lactogen, a key peptide hormone for regulating maternal metabolism and subsequent lactation [15,16].

**Figure 1:**
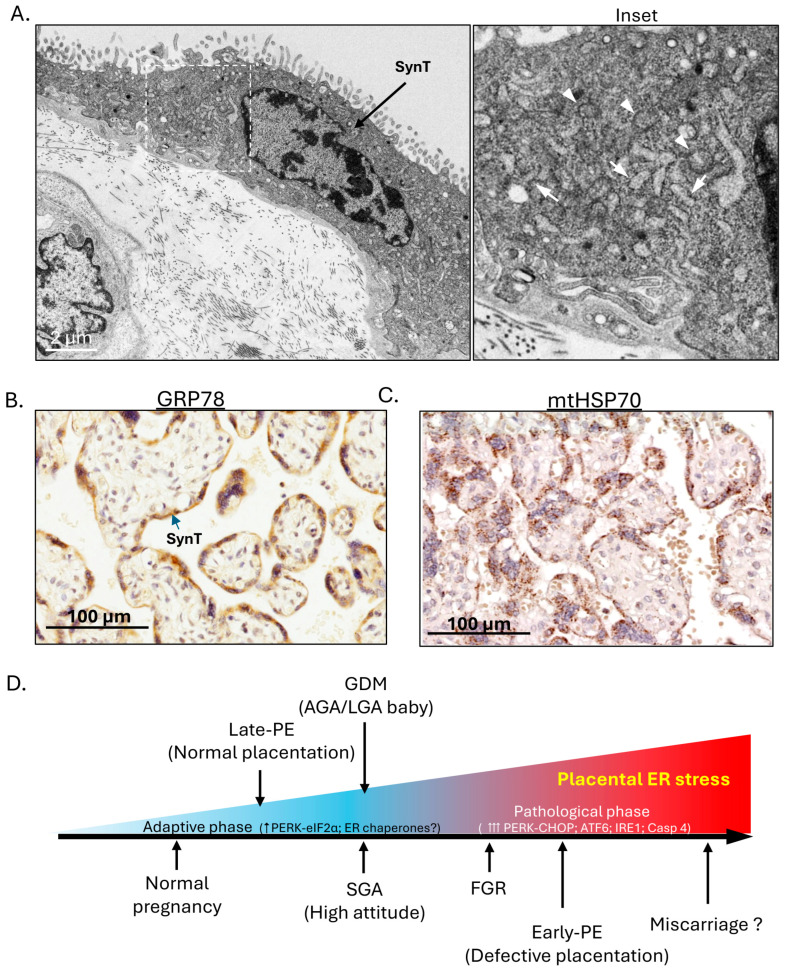
In the human placenta, the syncytiotrophoblast (SynT)/syncytial layer carries out both endocrine functions and nutrient exchange and potential placental ER stress severity spectrum across various complications of pregnancy. (**A**) Electron microscopy reveals a high density of ER cisternae and mitochondria in the syncytiotrophoblast/syncytial layer. Inset: Arrows indicate ER cisternae; arrowheads indicate mitochondria. (**B**,**C**) Immunohistochemical staining of human placental sections shows strong staining for ER chaperone GRP78 and mitochondrial chaperone HSP70, mainly in the syncytiotrophoblast/syncytial layer. (**D**) The placental ER stress severity spectrum chart summarises the severity of placental ER stress in various complications of pregnancy. (Remark: For (**A**–**C**), similar images have been published in our previous original research articles [20,21,23]).

This high rate of protein synthesis, along with the high energy demand necessary to support active transport of nutrients, makes the placenta highly susceptible to cellular stresses [17,18]. In the human placenta, the syncytiotrophoblast forms the epithelial covering of the chorionic villi and is bathed in maternal blood circulating in the intervillous space. It is here that nutrient and gaseous exchange takes place. It is also the primary site for placental protein and hormone synthesis. Electron micrographs show a high density of ER cisternae and mitochondria in the syncytiotrophoblast (SynT) (Figure 1A, Inset). These findings are confirmed by immunohistochemical staining of placental sections with antibodies raised against ER and mitochondrial chaperone proteins, heat shock protein 5A (HSP5A) (also known as glucose-regulated protein 78, GRP78) and GRP75 (also known as mitochondrial HSP70), respectively. These all show strong staining exclusively in the syncytiotrophoblast, consistent with its active endocrine function and high energy demand (Figure 1B,C). In pathological pregnancies, various cellular stresses affect the placenta, such as oxidative and nitrative stress, ER stress and mitochondrial stress. These are principally confined to the syncytiotrophoblast or syncytial layer [19,20,21,22].

## 3. Existence of ER Stress in Placentas from Various Complications of Human Pregnancy

ER stress, also known as the ER unfolded protein response (UPR^ER^), has been linked to the pathogenesis of various human pathologies, such as diabetes [24], neurodegenerative diseases [25] and cancer [26]. Our laboratory published the first molecular evidence of the existence of ER stress in placentas from complicated pregnancies, including fetal growth restriction (FGR) and early-onset pre-eclampsia (PE) [20]. In contrast, we did not observe increased stress in placentas from late-onset pre-eclampsia compared to normotensive controls [27]. Ultrastructural analyses of placentas from women with pre-existing diabetes [28] and pre-eclampsia demonstrated the presence of dilated ER cisternae [29], although the significance of the observations was not realised at the time. Excessively dilated cisternae are now recognised as a hallmark of ER stress. Using molecular markers, we subsequently confirmed the presence of placental ER stress in pregnancies complicated by GDM [30] and also observed low-grade ER stress in placentas from small-for-gestational age (SGA) pregnancies at high altitude [31]. In all studies we have only examined placentas delivered by elective caesarean section to avoid the confounding effects of labour. The maternal blood supply to the placenta is reduced during uterine contractions, and so labour causes a placental ischaemia–reperfusion type injury that itself induces ER stress [23]. We also ensured that our samples were collected and processed within 20 min of delivery to avoid postpartum ischaemic effects [32].

Interestingly, we observed a severity-dependent spectrum of ER stress in the placenta of these complicated pregnancies according to the molecular pathways of the ER stress response (ERSR) signalling being activated. To confirm this spectrum reflected severity, we used an ER stress inducer, tunicamycin, and performed a dose–response study in trophoblast-like cells. We observed sequential activation of all three ERSR signalling pathways as the dose and severity of ER stress increased [20]. Therefore, according to the temporal sequence of ERSR signalling pathways being activated, we could establish a potential placental ER stress severity spectrum across various complications of pregnancy. In normal pregnancy, there is a chronic low-grade ER stress in the murine placenta reflecting homeostatic adaptations to accommodate the high endocrine output [33], and it is likely that a similar situation exists in an apparently healthy human placenta at the end of an uncomplicated pregnancy. The ER stress severity spectrum starts from this low level, then increases in late-PE, SGA (at high attitude)/GDM (with AGA or LGA babies only), FGR and early-onset PE (Figure 1D). We speculate that the highest levels would be seen in cases of miscarriage when onset of the maternal circulation to the placenta is precious, as we have observed overwhelming related oxidative stress and dilated ER [34]. However, we have not tested these placentas specifically for markers of ER stress.

Furthermore, the existence of ER stress is not restricted to the placental tissues; increased levels of ER stress have been reported in maternal decidual tissues from cases of FGR and early-onset PE [35].

## 4. ER Stress Response (ERSR) Signalling Pathways

A variety of stress conditions, including glucose and amino acid deprivation, disruption of intracellular calcium homeostasis and inhibition of cellular glycosylation, were initially identified as promoting the accumulation of “malfolded” proteins in the ER cisternae and inducing expression of the ER resident chaperones GRP78 and GRP94 [36]. It is now appreciated that the ER contains a highly sophisticated quality-control system that regulates protein homeostasis, or “proteostasis”. This system involves the inhibition of protein translation, stimulation of ER-associated protein degradation (ERAD) and increasing protein chaperones and autophagy [37]. In response to dysregulated proteostasis, the accumulation of misfolded or unfolded proteins in the ER activates the “adaptive phase” of ERSR signalling pathways that aim to restore ER protein homeostasis [38]. However, when the stress condition is persistent and causes irreversible damage to the cells, the “pathological phase” of ERSR signalling pathways is activated to induce apoptosis and eliminate the cells [38].

ERSR signalling pathways consist of three highly evolutionarily conserved pathways: the eukaryotic translation initiation factor 2α kinase 3 (also known as protein kinase RNA (PKR)-like ER kinase or PERK), activating transcriptional factor 6 (ATF6) and inositol requiring enzyme 1 (IRE1) pathways [38]. In the adaptive phase, the ERSR signalling pathways attenuate protein translation, activate expression of ER chaperones, increase anti-oxidant defences and promote ERAD, thereby attempting to restore proteostasis in the ER. In the pathological phase, the same pathways additionally inhibit anti-apoptotic pathways and activate caspases, thereby promoting cell death [39].

## 5. Causative Factors That May Induce Placental ER Stress in Complicated Pregnancies

Extrinsic factors such as hypoxia, amino acid deprivation, glucose deprivation and viral infection activate ERSR signalling pathways [40,41,42,43], and some are likely to be causative in the mild activation seen, for example, in GDM and SGA at high altitude. Exposure of trophoblast cells to either high glucose or an hypoxic environment induces low-grade ER stress, in which only the PERK pathway is activated, consistent with the in vivo findings in which the dilation of ER cisternae in syncytiotrophoblast is mild [30,31]. However, the high glucose concentration alone does not directly activate ER stress. Instead, the high glucose induces metabolic acidosis, which is particularly prominent in the metabolically active villous trophoblasts, which are particularly vulnerable. This in turn mediates ER stress activation [30].

In the majority of cases of both FGR and early-onset PE [44,45], there are no obvious extrinsic causative factors. However, defective placentation due to insufficient transformation of the maternal uterine spiral arteries that supply the placenta commonly plays a central role in the pathogenesis of both complications [46,47]. In normal pregnancy, the spiral arteries undergo extensive remodelling during early pregnancy in order to allow a large volume of maternal blood to flow into the placental intervillous space (IVS) at low velocity and pressure [48]. This arrangement provides a rich source of nutrients and oxygen for maternal–fetal exchange without causing damage to the delicate placental villous trees. The remodelling is mediated by extravillous trophoblast (EVT) cells that migrate from the placental villi into the wall of the uterus beneath the placental attachment site and involves the loss of smooth muscle from the arterial walls and dilation of the vessel mouths. In contrast, deficient remodelling leads to the persistence of vasoreactivity due to the retention of vascular smooth muscle cells in the vessel walls and a lack of dilatation. The result is that pulsatile maternal blood flow at high resistance and velocity is maintained, resulting in a “jet-like” blood flow into the IVS. This causes physical damage to the fragile placental terminal villi, creating villus-free cavities and exposing the syncytiotrophoblast to high shear stresses [48]. Crucially, the persistence of vasoreactivity results in intermittent blood flow into the IVS, inducing ischaemia–reperfusion-type injury and causing oxidative damage to the placenta. The “Great Obstetrical Syndromes” are now well recognised by clinicians to be closely associated with defective deep placentation [49], and placental oxidative stress plays a detrimental role in their pathophysiology [50,51]. Oxidative stress is closely interlinked with ER stress due to the intimate connections between mitochondria and ER cisternae, and it is likely that the two stresses occur in tandem [52].

Evidence to support this scenario comes from the in vitro repetitive hypoxia–reoxygenation (rHR) culture model, which mimics intermittent blood flow into the placenta and induces persistent ER stress in BeWo trophoblast-like cells. The severity of the rHR correlates with the degree of ER stress induced, as assessed by the sequential activation of PERK, ATF6 and IRE1 signalling pathways [27]. Furthermore, ER stress is induced in the placenta during labour, when it is known that the maternal arterial supply is reduced during uterine contractions, causing an rHR insult in vivo [23,53]. Finally, infection of pregnant women with Zika virus can lead to FGR or even stillbirths, and evidence indicates that these outcomes are mediated by ER stress [54]. Exposure of placental cells to Zika virus activates both the PERK and IRE1 arms of the ERSR signalling pathways and induces apoptosis [55].

## 6. Impact of Placental ER Stress on Pregnancy Outcome

ER stress has the potential to modulate the function and differentiation of trophoblast subtypes at different stages of pregnancy, including the differentiation of EVT and their invasion into the uterine wall, thereby modulating transformation of the maternal spiral arteries [56,57]. During EVT differentiation the cells undergo an epithelial–mesenchymal transition (EMT), and this represents a key step in their acquisition of the invasive phenotype [58]. Crucially, several transcription factors regulating EMT such as SNAL1, SNAI2, ZEB2 and TCF3 are direct targets of XBP1, and activation of the IRE1-XBP1 pathway promotes EMT [56]. In addition, matrix metalloproteinase 2 is central for EVT invasion and its transcription and translation are negatively regulated by ATF4 and eIF2α (which function in the PERK pathway) [57]. ER stress may therefore impair EVT invasion, but whether it plays a beneficial or detrimental role in vivo remains unclear.

The high metabolic rate of the syncytiotrophoblast exposes organelles such as the mitochondria to constant oxidative damage, leading to their turnover and the need for replenishment by cytotrophoblast fusion [59,60]. All three ERSR signalling pathways are activated during fusion and syncytialisation of cytotrophoblast cells [61]. Therefore, the combination of continual cytotrophoblast fusion and the high secretory nature of the syncytiotrophoblast may contribute to the basal low-grade ER stress observed in placentas from normal pregnancies.

On top of this background level, the further activation of ERSR signalling pathways seen in pregnancies complicated by GDM or by hypobaric hypoxia at high altitude serves to maintain ER homeostasis under unfavourable conditions. In doing so it may lead to a degree of translational arrest, slowing placental cell proliferation and resulting in the smaller placenta typically associated with SGA babies [62]. Indeed, a sublethal level of ER stress reduces cell proliferation in trophoblastic-like cells [20]. At the opposite end of the ER stress spectrum, activation of all three ERSR signalling pathways, including upregulation of the pro-apoptotic transcriptional factor CHOP and activation of caspase 4, is seen in early-onset PE [20,63]. In these cases, there will be additional activation of apoptotic and senescence pathways, particularly in the syncytiotrophoblast [64]. Senescence is associated with adoption of the senescence-associated secretory phenotype, and the pro-inflammatory cytokines secreted, including interleukin-1 (IL-1), IL-6 and IL-8, may contribute to development of the peripheral syndrome by activating the maternal endothelial cells. Finally, excessive shedding of syncytiotrophoblast microvesicles and debris are observed mainly in early-onset PE, but not in FGR [65]. This difference correlates with the finding that the release of microvesicles from trophoblast-like cells in vitro is stimulated by high levels of ER stress [66]. As these microvesicles carry miRNAs, DNA and lipids, they have been implicated in the development of the peripheral syndrome of pre-eclampsia [45,67,68].

## 7. Importance of Animal Models in Pregnancy Research

Direct analysis of pathological placental and uterine tissues, body fluids such as plasma/sera/urine from the patients [69,70] and non-invasive imaging such as magnetic resonance [71] and ultrasonography [72] have undoubtedly enhanced our understanding of the pathophysiology of complications of pregnancy. However, due to the lack of any opportunity for manipulation, the majority of results can only be interpreted as correlative and have a limited role in revealing the molecular mechanisms underpinning the pathology and its progression. Additionally, a healthy pregnancy outcome is reliant on a harmonious three-way communication among mother, placenta and fetus. This cannot be recapitulated, even by the most sophisticated in vitro systems or models.

Many species of animals have been used for studying different aspects of human pregnancy, including normal embryonic and placental development and investigating the pathogenesis of pregnancy complications [73,74]. The most common include sheep, guinea pig, rat and mouse. Non-human primates, such as chimpanzees, baboons and rhesus macaques, share similar physiological, genetic and developmental pregnancy stages with humans and were considered “gold standard” for human pregnancy research in the past [75,76,77]. However, their use now is largely prohibited or limited in many countries because of increasing ethical concerns around their high cognitive capacity [78].

Due to the great variation in body size, gestational length and types of placenta among species, each species has its own strengths and weaknesses in terms of physiological relevance and practicality. In addition, it must be recognised that many complications of pregnancy, such as pre-eclampsia, are virtually restricted to humans. Although hypertension may be induced during pregnancy in animal models, the full peripheral syndrome does not occur spontaneously. The molecular biological reagents and sequencing resources available also vary widely, and therefore, choosing the right animal model is dependent on the kind of scientific questions being asked.

Sheep are widely used for understanding fetal physiological changes in normal and FGR pregnancies [79], as the fetus shows similar developmental milestones to the human fetus, as well as similar rates of fetal oxygen consumption [80,81], placental glucose transfer and metabolism [82,83]. Short-term exposure of pregnant sheep at late gestation to a hypoxic environment (10% O_2_) recapitulates some of the physiological and molecular markers of pre-eclampsia in the mother, placenta and offspring [84]. Consistent with findings in the human placenta at high altitude, the hypoxic ovine placenta shows low-grade ER stress [84]. However, maintaining sheep is expensive and requires specialist facilities.

**Figure 2:**
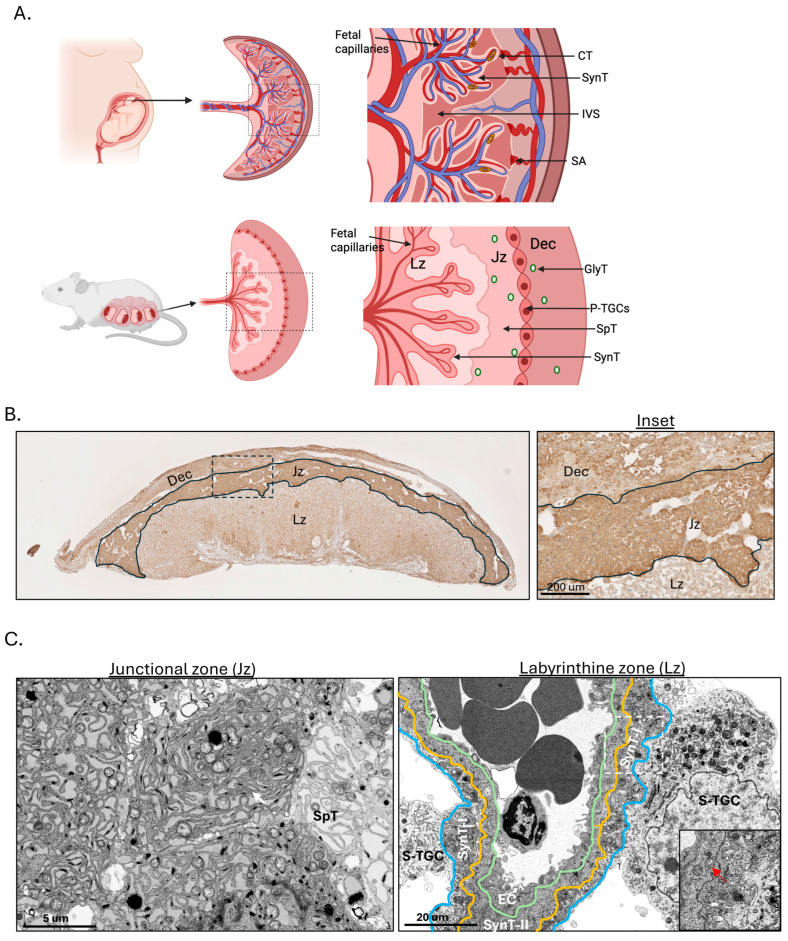
An anatomical comparison between human, functional differences and trophoblast subtypes in different region of murine placenta. (**A**) An illustration showing anatomical comparison between human and murine placenta. (**B**) Immunohistochemical analysis shows strong staining for the ER chaperone Grp78 mainly in the Jz region. Inset shows close up view of staining in 3 placental regions. (**C**) Electron microscopic analysis reveals a high density of ER cisternae present in the spongiotrophoblasts in the Jz, while there are only scattered ER cisternae in the syncytiotrophoblast in the Lz. White arrow indicates ER cisternae and red arrow indicates mitochondrion. Inset shows mitochondria, but not ER cisternae in both SynT I and II. Dec—decidua; Jz—Junctional zone; Lz—Labyrinthine zone; S-TGC—Sinusoidal trophoblast giant cell; SynT l—Syncytiotrophoblast I; SynT ll—Syncytiotrophoblast ll; EC—Endothelial cell; CT—Cytotrophoblast; SynT—Syncytiotrophoblast; IVS—Intervillous space; SA—Spiral artery; GlyT—Glycogen trophoblast; SpT—Spongiotrophoblast; P-TGC—Parietal trophoblast giant cell. Created in BioRender. Yung, H.W. (2026) https://BioRender.com/sp6jflu (Remark: The placentas used for the above images were from homozygous *floxed Xbp1* (*Xbp1^fl/fl^*) animals, in which two *loxP* sites are located in introns; therefore, the phenotype is equivalent to the wild-type animal. Similar images have been published in our previous original research articles [86,89]).

Rodents are relatively small in size and have short gestations, 20–22 days and so husbandry is easier. Additionally, the rodent placenta is discoid and haemochorial, similar to the human placenta. Importantly, in the rodent placenta transport and endocrine functions that in the human are performed by the syncytiotrophoblast are separated into two different compartments, the labyrinth and junctional zones, respectively [85] (Figure 2A). Consequently, the murine syncytiotrophoblast within the labyrinth contains little ER, whereas the spongiotrophoblast cells of the junctional zone have a dense complement (Figure 2B,C, Inset). This distinctive feature makes the rodent placenta a powerful tool for manipulating endocrine pathways without affecting transport functions, and vice versa [86]. Other advantages of the rodent placentas for studying transplacental nutrient transport mechanisms in FGR were reviewed by Winterhager and Gellhaus [87]. Genetic manipulation of the rat has been found to be more challenging than that of the mouse [88], and hence the majority of genetically manipulated transgenic rodent models are murine.

## 8. Transgenic ER Stress Mouse Models Reveal the Role of the Ire1 Pathway in Placental Development

To investigate molecular pathophysiological mechanisms in a disease, it is important to be able to manipulate the expression of specific gene(s), positively or negatively, in vivo. However, in the traditional transgenic mouse model, regardless of whether it is a gene knockout (a global deletion of the gene) or knock-in of a transgene (a global expression of the transgene), there is one obvious disadvantage, that genetic manipulation affects all cell types in the animal. As a result, the changes in cell type/tissue/organ of interest in the transgenic animals could be confounded by changes in other cell types/tissues/organs. In a large-scale phenotyping study of 103 transgenic mouse models, 25–30% of gene knockouts had placentation defects and intrauterine lethality [90]. Therefore, several novel techniques have been developed which allow tissue-specific genetic manipulation. Three systems are now widely adopted: the Cre/LoxP system [91], transposase [92] and endonuclease-based gene modification (e.g., CRISPR/Cas9 system) [93].

Many transgenic mouse models targeting ERSR signalling pathways have been created, and the 16 most common, along with their pregnancy outcomes, are summarised in Table 1. These include 12 global gene knockouts, 2 fetus-specific gene knockouts, 1 placental junctional zone-specific gene knockouts and 1 with site-directed mutagenesis. Unfortunately, studies using many of these models did not report data relating to fetal and/or placental growth and development. Nevertheless, they still provide valuable insight into the roles of different ERSR signalling pathways during pregnancy.

In brief, either deletion or mutation of genes in the Perk pathway has limited impact on embryonic survival, with the majority of transgenic newborns viable but dying prematurely at the postnatal stage due to pancreatic dysfunction [94,95] (Table 1). In the Atf6 pathway, deletion of either *Atf6α* and *Atf6β* alone does not cause embryonic lethality and shows no adverse effects. However, the deletion of both *Atf6α* and *Atf6β* is embryonic lethal [96,97]. However, a single copy of either *Atf6α* or *Atf6β* gene can partially rescue the phenotype [97]. Crucially, a fetal-specific deletion of both *Atf6α* and *Atf6β* is also embryonic lethal [98]. These observations indicate there is some redundancy and that the Atf6 pathway is crucial for fetal growth and development. Finally, in the Ire1 pathway, deletion of either *Ire1α* or *Xbp1* causes embryonic lethality at embryonic ages 13.5 and 14.5, respectively [99,100]. No phenotype was observed in *Ire1β*^−/−^ animals [101]. Thus, Ire1β does not compensate for loss of function of Ire1α [101]. However, following fetal-specific deletion of *Ire1α*, all pups are born viable and at the expected Mendelian ratio, and this shows that the Ire1α pathway plays a crucial role in placental development, rather than affecting fetal growth and development [33].

**Table 1:** List of ERSR pathway transgenic mouse model and the pregnancy outcomes.

Transgenic Model	Genetic Manipulation	Pregnancy Outcomes ^#^	Reference
**PERK Pathway**			
*Eif2ak3*^−/−^ (*Perk*^−/−^)	Global *Perk* knockout	- Partial embryonic lethality (Below expected Mendelian ratio at birth - About a quarter of newborns die within first 48 h - No fetal growth restriction - Exhibits severe postnatal growth retardation - Early mortality - No information on placenta	[94,95]
*Tpbpa^+/Cre^.Perk* ^−/−^	Placental Jz-specific *Perk* knockout by crossing *Tpbpa.Cre* animal with *Perk^fl/fl^* animal	- Expected Mendelian ratio at birth - No fetal growth restriction - Disrupted maternal hepatic glucose metabolism	[86]
*Eif2s1^A/A^* (*eIF2α^A/A^*)	Site-directed mutagenesis at residue 51 from serine to alanine	- Expected Mendelian ratio at birth - Majority of mutant newborns die within first 18 h - Fetal growth restriction in both placenta and fetus - Diminished exchange capacity in placental labyrinth zone - Accumulates misglycosylated protein aggregates in placental junctional zone - Reduced trophoblast stem cell and differentiated trophoblast population - Reduced syncytiotrophoblasts, glycogen cells and trophoblast giant cells’ population	[89,102,103]
*Atf4* ^−/−^	Global *Atf4* knockout	- Expected Mendelian ratio at E17.5 - Majority of mutants die within first hour after birth - Fetal growth restriction at E15.5. - Displays severe postnatal growth retardation - No information on placenta	[104,105]
*Ddit3*^−/−^ (*Chop*^−/−^)	Global *Chop* knockout	- Expected Mendelian ratio at birth - Normal postnatal growth	[106]
*Nfe2l2*^−/−^ (*Nrf2*^−/−^)	Global *Nrf2* knockout	- Expected Mendelian ratio at birth - Fetal growth restriction in both placenta and fetus from E15.5 - Diminished exchange capacity in labyrinth zone volume in placenta	[107,108]
*PPP1R15A*^−/−^ (*Gadd34* ^−/−^)	Global *Gadd34* knockout	- Expected Mendelian ratio at birth - Normal postnatal growth and development - No information on fetus and placenta	[109]
*P58ipk* ^−/−^	Global *p58ipk* knockout	- Expected Mendelian ratio at birth - Displays postnatal growth retardation - Early mortality at about 14.5 months	[110]
**ATF6 pathway**			
*Atf6α* ^−/−^	Global *Atf6α* knockout	- Expected Mendelian ratio at birth - No information on fetus and placenta	[96,97]
*Atf6β* ^−/−^	Global *Atf6β* knockout	- Expected Mendelian ratio at birth - No information on fetus and placenta	[96]
*Atf6α* ^−/−^ *.Atf6β* ^−/−^	Global double *Atf6α* and *Atf6β* knockout	- Global embryonic lethality - No information on fetus and placenta and the gestation age of lethality	[97]
*Mox2^+/Cre^.Atf6α^ΔZIP/ΔZIP^.Atf6β^ΔZIP/ΔZIP^*(*Fetal-specific Atf6α*^−/−^*.Atf6β*^−/−^)	Fetal-specific double *Atf6α* and *Atf6β* knockout by crossing *Mox2.Cre* animal with double *Atf6α^fl/fl^* and *Atf6β^fl/fl^* knockout animal	- Global embryonic lethality - No information on fetus and placenta and the gestation age of lethality	[98]
***IRE1 pathway***			
*Ern1*^−/−^ (*Ire1α* ^−/−^)	Global *Ire1α* knockout	- Embryonic lethality at E13.5 - Fetal growth restriction of both fetus and placenta at E12.5 - Diminished exchange capacity in placental labyrinth zone	[33,100]
*Mox2^+/Cre^.Ire1^αΔNeo/ΔR^*(*Fetal-specific Ire1*^−/−^)	Fetal-specific *Ire1α* knockout by crossing *Mox2.Cre* animal with *Ire1^fl/fl^* knockout animal	- Newborns are viable - Expected Mendelian ratio at birth - Normal fetal and placental weight	[33]
*Ern2*^−/−^ (*Ire1β*^−/−^)	Global *Ire1β* knockout	- Expected Mendelian ratio at birth - Normal postnatal growth - No information on fetus and placenta	[101]
*Xbp1* ^−/−^	Global *Xbp1* knockout	- Embryonic lethality at E14.5 - Fetal growth restriction with smaller fetus and placenta - Diminished exchange capacity in placenta labyrinth zone.	[33,99]

Remarks: ^#^ The consequence of the deletion of the target gene is only focused on the pregnancy outcome, not the impact on other tissues or organs.

To summarise, the pregnancy outcomes from these transgenic animal models of ERSR signalling pathways revealed distinctive functions of each arm of ERSR signalling pathways in fetal and placental development. The PERK pathway likely acts as a homeostatic response to ensure survival of the fetus in an unfavourable environment. This is consistent with our observations in human SGA pregnancy at high attitude and GDM [30,31]. In contrast, the ATF6 pathway plays a crucial role in the regulation of fetal development, but its role in placental development remains unclear. Finally, the IRE1 pathway is essential in the development of the placenta but not of the fetus.

## 9. Use of Models of ER Stress in Deciphering Pathophysiological Roles of Placental ER Stress in Pregnancy Complications

The placentas from *Ire1α*^−/−^ and *Xbp1*^−/−^ mutants show similar defective vascular development in the labyrinth zone, supporting the potential role of the IRE1 pathway in placental angiogenesis [33]. Indeed, expression of the key angiogenic growth factor, vascular endothelial growth factor (VEGFA), is regulated by IRE1α-XBP1 [111]. Defective development of the labyrinth zone restricts nutrient supply and gas exchange to the fetus, impairing its growth and, if sufficiently severe, ultimately leading to its death. In addition, expression of another angiogenic factor, placental growth factor (PlGF), which modulates angiogenesis under pathological conditions, is partially regulated by ATF4 and ATF6β in placental cells [112]. Nevertheless, ER stress-mediated endothelial dysfunction is recognised as a key step towards the onset of atherosclerosis and cardiovascular diseases [113]. It is also a contributor to both placental and maternal vascular pathology in pre-eclampsia. Furthermore, the expression of several members of carcinoembryonic antigen-related cell adhesion molecule (CEACAM) and pregnancy-specific glycoprotein (PSG) families are also directly regulated by the IRE1α-XBP1 pathway [114]. Although the exact role of CEACAMs and PSGs in pregnancy remains elusive, they may act as immunomodulators [115,116]. Indeed, decreased PSG levels in maternal blood are reported in multiple pregnancy complications [116]. Therefore, placental ER stress may contribute to the maternal peripheral symptoms in early-onset PE through modulation of expression of angiogenic factors and immunomodulators (PSGs and CEACAMs).

The phosphoinositide-3-kinase-protein kinase B/Akt-mammalian target of rapamycin (PI3K-Akt-mTOR) is a core signalling pathway in the regulation of cellular metabolism, cell survival, growth and proliferation [117,118]. The pathway is active in the placenta [119] and is down-regulated in pregnancies complicated by FGR and early-onset PE [20,27]. The loss of AKT signalling is due to both reduced kinase phosphorylation and lower total protein levels, which is due to translational inhibition, in those placentas [20]. Insulin-like growth factors (IGF1 and IGF2), which activate PI-3K-AKT-mTOR pathways [120], are two major growth factors promoting placental and fetal growth [121,122]. The placenta produces IGFs that can also act locally [123].

To consolidate potential causal linkages between ER stress and the loss of AKT-mTOR signalling observed in pathological placental tissues and in vitro, a transgenic ER stress model with no embryonic lethality is required. The PERK pathway initiates the first line of defence upon ER stress and is mediated by phosphorylation of the alpha subunit of eukaryotic initiation factor 2 (eIF2α) at the regulatory serine residue at position 51, which in turn attenuates protein translation [43]. In the *eIF2α^A/A^* (or *Eif2s1^A/A^*) animals, serine at position 51 has been mutated to alanine by site-directed mutagenesis; therefore, protein translation inhibition induced by all upstream kinases is blocked, resulting an increased basal protein translation rate [102]. The placenta displays a basal level of ER stress even under normal physiological conditions, which is likely homeostatic [33]. Indeed, the excessive high basal protein translation in the *eIF2α^A/A^* mutant induces ER stress exclusively in the junctional zone (Jz) of the placenta [89]. Importantly, both the fetus and placenta of *eIF2α^A/A^* mutants show a FGR phenotype and the smaller placenta [89].

In the *eIF2α^A/A^* mutant placenta, Akt phosphorylation is decreased in both the Jz and Lz, while total Akt protein is only reduced in the Jz, coexisting with the ER stress [89], consistent with ER stress-mediated AKT translation inhibition [124]. Interestingly, the *eIF2α^A/A^* mutant MEFs, which also display ER stress, secrete mainly pro-Igf2, and this may explain the reduced Akt phosphorylation in the mutant placenta [89]. In animals with deletion of *Akt1*, the progeny also exhibit growth restriction, with smaller placentas as early as E14.5 [125]. This confirms the importance of the AKT signalling pathway for placental growth. Furthermore, the ability of AKT to recognise its downstream substrates depends on the phosphorylation status of the Ser473 residue [126,127]. In response to ER stress, GRP78 is increased and binds to AKT, blocking Ser473 phosphorylation and thereby modulating downstream substrate specificity [128]. Nevertheless, the results from the *eIF2α^A/A^* animal support the hypothesis that ER stress-mediated down-regulation of AKT signalling in the placenta plays a key role in the pathogenesis of FGR.

Furthermore, in the *eIF2^A/A^* mutant placenta, despite the absence of overt ER stress, Lz volume was reduced, compromising placental nutrient exchange [89]. *eIF2α^A/A^* blastocysts at E3.5 and placenta at E9.5 have developmental delay and reduced the trophoblast stem cell population in the trophectoderm layer [103]. Therefore, ER stress-mediated differentiation of TSCs diminished the trophoblast stem cell pool, resulting in smaller placentas, while ER stress perturbs trophoblast stem cell differentiation into specific trophoblast lineages, resulting in placental dysmorphogenesis [89,103]. In the human, activation of all three ERSR signalling pathways is accompanied by differentiation of the cytotrophoblast [61]. Inhibition of ATF6α, IRE1α or PERK alone is sufficient to reduce cell fusion in the primary cytotrophoblast [61]. However, only the inhibition of ATF6α and IRE1α, but not PERK impaired hCG secretion by the cells, indicating the complexity of ERSR signalling pathways in cytotrophoblast fusion, differentiation and syncytiotophoblast function. Additionally, protein kinase A (PKA), which facilitates cytotrophoblast fusion upon activation, also phosphorylates IRE1α on residue Ser 724, which is crucial for its enzymatic activity [129,130]. These observations may imply that the co-activation of ERSR signalling pathways during trophoblast syncytialisation enhances protein folding capacity in the differentiated syncytiotrophoblast to cope with its high secretory nature.

To summarise, studies using the *Ire1α*^−/−^, fetal-specific *Ire1α*^−/−^, and *eIF2α^A/A^* transgenic models reveal the molecular mechanisms by which ER stress contributes to the pathogenesis of FGR in the placenta and subsequently the fetus. The equivalent findings of ER stress in negative regulation of PI-3K-AKT signalling pathways in the smaller placentas seen in human FGR and *eIF2α^A/A^* mutant mouse supports the relevance of findings in the mouse for human disease. However, there are significant differences in terms of trophoblast subtypes and the anatomical structure of the placenta between the mouse and human, and so the findings on ER stress and trophoblast differentiation must be interpreted with caution.

## 10. Placental Endocrine-Specific ER Stress and Maternal Long-Term Health After Complicated Pregnancies

The impact of pregnancy complications on both maternal and fetal health extends beyond pregnancy by increasing the risk of subsequent development of cardio-metabolic diseases [4,5]. The Development Origins of Health and Disease (DOHaD) theory, also known as the “Barker hypothesis”, states that an unfavourable intra-uterine environment may lead to epigenetic and structural changes in fetal tissues and organs, thereby programming babies for chronic disease in adulthood [131]. In contrast, the mechanisms rendering women at higher risk of developing cardio-metabolic diseases after complicated pregnancies are largely unexplored. One of the reasons is the lack of an animal model that recapitulates the biological changes on the maternal side of a complicated pregnancy.

Using the Cre-LoxP system, several placental tissue- or trophoblast-specific Cre transgenic animals have been created, including *Elf5.Cre* (all trophoblast lineages) [132]; *Tpbpa.Cre* (spongiotrophoblasts in junctional zone) [133]; *CYP19.Cre* (trophoblasts in labyrinthine zone) [134]; and *Gcm1.Cre* (syncytiotrophoblast II) [135]. These new Cre models allow for specific genetic manipulations targeting a cellular process/gene function, a specific trophoblast subtype, placental compartment/zone or the entire placenta. These models are particularly valuable for studying how the placenta modulates fetal growth and development, as well as maternal physiological adaptations, in normal and complicated pregnancies.

Emerging evidence from our laboratory reveals that placental ER stress may modulate maternal physiological adaptations during pregnancy and beyond by affecting protein glycosylation [86]. Glycosylation is one of the core post-translational modification processes and is initiated in the ER lumen and completed in the Golgi apparatus. It is crucial for protein function and activity as it regulates aspects such as protein circulating half-life, solubility, interaction with receptor/other proteins and immune recognition [136]. Indeed, ER stress not only inhibits end-cap sialyation, but it also promotes oligomannose synthesis necessary for the glycosylation of secreted glycoproteins [86]. It is known that the loss of the sialic acid end-cap or having a high mannose N-glycan structure, or both, leads to rapid clearance by the hepatic asialoglycoprotein receptor (ASGR) and mannose receptor (Mrc1) [137,138,139]. Interestingly, this process is particularly prominent in reproduction because progesterone is one of the key regulators of the expression of these two receptors [137]. A double knockout of both *Asgr2* and *Mrc1* genes promotes a remarkable accumulation of PSGs and CEACAM protein in maternal circulation [138].

Upon ER stress, cells, including placental cells, produce and secrete misglycosylated proteins, potentially with reduced bioactivity [86,140]. This phenomenon is observed in early-onset PE, when the placenta secretes misglycosylated glycoproteins (e.g., pregnancy-specific glycoprotein 5, PSG5) into the maternal circulation [86]. Placental hormones and proteins mediate maternal adaptation to support pregnancy and lactation [14], and hence their loss or a reduction in their bioactivity will likely impact adversely on maternal health.

As shown in Figure 2B,C, the spongiotrophoblast, the main cell type in the Jz, contains vast quantities of ER cisternae and stains strongly for the ER chaperone Grp78. We generated a placental Jz-specific ER stress model, *Jz-Perk*^−/−^, in which *Perk* is specifically deleted in the Jz by crossing *floxed Perk* (*Perk^fl/fl^*) and *Tpbpa.Cre* animals. This perturbs the secretion of ER stress-mediated misglycosylated placental factors into the maternal circulation [86]. Despite the existence of low-grade ER stress in the Jz in normal pregnancy [33], the deletion of *Perk* fails to further activate ER stress in the Jz [86] but does render *Perk*^−/−^ cells hypersensitive to additional stresses [141]. One such stress is reduced oxygen, which in wild-type mice only activates the Perk arm of the ERSR signalling pathways [142]. Exposure of female carrying *Jz-Perk*^−/−^ mutant placentas to reduced oxygen induces ER stress in the Jz, causing protein misglycosylation exclusively in the Jz [86]. Despite no change in placental and fetal weights, dams carrying a litter of *Jz-Perk*^−/−^ mutant placentas exhibit abnormal maternal hepatic glucose metabolism, increased hepatic cellular stress and depleted hepatic energy storage [86]. Intriguingly, these livers show increased expression of hepatic DNA methyltransferase (Dnmt3a) [86], one of the key enzymes that facilitates de novo DNA methylation in somatic cells [143]. This observation suggests a linkage between placental ER stress and the postpartum risk of metabolic diseases in women after complicated pregnancies.

## 11. Targeting Placental ER Stress: A Potential Therapeutic Intervention for Pregnancy Complications?

Advances in our understanding of the contribution of ER stress to the pathophysiology of human diseases has led to the rapid development of therapeutic interventions aiming to alleviate ER stress or to inhibit specific protein/kinase in the ERSR signalling pathways. Indeed, some of these are already in preclinical and clinical trials for diseases such as cancer, diabetes and neurodegenerative diseases [144,145]. For example, a GRP78 inhibitor (IT-139) was in a phase 1 clinical trial for advanced solid tumours [146]. In comparison to other diseases, drug use for treating pregnancy complications requires a particularly stringent scrutiny to ensure its safety for both the mother and the developing fetus. Among all ER stress inhibitors, UDCA (urodeoxycholic acid) and its derivative, TUDCA (Tauroursodoxycholic acid) [147], are produced naturally in low quantities by the liver and secreted in bile. Hence, they are two potential chemical chaperones for alleviating placental ER stress in pregnancy. The beneficial effects of TUDCA operate at multiple levels. It acts a chaperone binding to and stabilizing unfolded proteins, reduces the activation of the UPR sensors by inhibiting the dissociation of GRP78 and blocks the activation of downstream pro-apoptotic pathways. It can also increase resistance to oxidative stress and mitochondrial dysfunction by stimulating levels of the mitochondrial anti-oxidant enzyme manganese superoxide dismutase (MnSOD) [148]. UDCA is already used to treat intrahepatic cholestasis of pregnancy, with a good safety record [149,150,151]. Although no reduction in adverse perinatal outcomes, including preterm birth, perinatal death or admission to a neonatal intensive care unit, was found [149], a significant increase in birthweight and trend for a reduced incidence of GDM has been reported [150]. Our laboratory also demonstrated that administration of TUDCA in the drinking water during pregnancy in *eIF2α^S/A^* mutant mice reduced ER stress in the *eIF2α^A/A^* placenta and reduced the number of resorptions and mutant embryos with developmental delay [103]. Administration of TUDCA to pregnant aged mice has been shown to restore pup birthweight, reduce markers of ER stress in the placenta and improve maternal vascular function [152,153]. In addition, supplementation with TUDCA improves embryo quality and survival rate during in vitro-produced bovine embryos [154]. These observations suggest potential beneficial actions of UDCA or TUDCA in improving pregnancy outcome under pathological conditions, but further safety tests are required given their pleiotropic effects. Recent developments, such as the derivation of human trophoblast organoids [7,155], open new avenues for addressing these concerns at the cellular, but not the whole organismal, level.

Finally, although there are many small molecule inhibitors and peptide inhibitors targeted specific to PERK, ATF6, IRE1 and their downstream effectors, they have been largely developed or tested for cancers, immune diseases and neurodegenerative disorders [156,157]. The use of these drugs in pregnancy will need extensive research to evaluate their safety, although sophisticated placental-targeted delivery systems offer new potential [158]. Furthermore, as ER stress is also part of the physiological homeostatic response, targeting stress to these pathways may provoke harmful, rather than beneficial effects [159] unless the correct dosage is used. Due to the heterogeneity of early-onset PE and FGR, any therapeutic interventions aimed at alleviating ER stress require considerable caution.

## 12. Conclusions

The placenta is a major endocrine organ, secreting large quantities of polypeptide and steroid hormones that mediate maternal adaptations to pregnancy. Consequently, it is susceptible to ER stress, but in comparison with other organs, little attention has been paid to the role of ER stress in pregnancy complications. Nevertheless, murine transgenic models reveal an essential role of the PERK arm of the ERSR signalling pathways in ensuring survival of the fetus under unfavourable conditions. Activation of this pathway in both human pregnancies at high attitude and murine pregnancies housed under hypoxic conditions homeostatically matches placental and fetal growth to oxygen and nutrient availability [31,142]. By contrast, activation of the ATF6 and IRE1 pathways in pregnancies associated with severe placental malperfusion contributes to additional changes, including impaired mitochondrial activity, increased autophagy, release of microvesicles and placental senescence. The microvesicles and pro-inflammatory cytokines released may cause activation of the maternal endothelium and the peripheral syndrome of pre-eclampsia. Finally, ER stress may compromise the bioactivity of placental peptide hormones through mis-glycosylation, impairing maternal adaptation to pregnancy. The structure of the murine placenta with separate transport and endocrine zones which can be manipulated individually using transgenic technologies enables the impact of ER stress on different placental functions to be assessed independently. Equally, transgenic models enable a new era for exploring how placental dysfunction programmes maternal organs/tissues for increased risk of cardio-metabolic diseases following a complicated pregnancy.

## Data Availability

We confirm that similar but not identical images in Figure 1A–C and Figure 2 have been published in our previous original research articles. No new data were created or analysed in this study.

## Abbreviations

The following abbreviations are used in this manuscript:

ER	Endoplasmic reticulum
FGR	Fetal growth restriction
PE	Pre-eclampsia
GDM	Gestational diabetes mellitus
AGA	Appropriate for gestational age
LGA	Large for gestation age
ERSR	ER stress response
UPR^ER^	ER Unfolded protein response
GRP78	Glucose regulated protein 78
PERK	Protein kinase RNA (PKR)-like ER kinase
ATF6	Activating transcriptional factor 6
IRE1	Inositol requiring enzyme 1
IVS	Intervillous space
rHR	Repetitive hypoxia–reoxygenation
PSG	pregnancy-specific glycoprotein
CEACAM	Carcinoembryonic antigen-related cell adhesion molecule
IGF	Insulin-like growth factor
eIF2α	Eukaryotic initiation factor 2 subunit alpha
Jz	Junctional zone
Lz	Labyrinthine zone
Dec	Decidua
UDCA	Urodeoxycholic acid
TUDCA	Tauroursodoxycholic acid
